# Adaptation of *Lymnaea fuscus* and *Radix balthica* to *Fasciola hepatica* through the experimental infection of several successive snail generations

**DOI:** 10.1186/1756-3305-7-296

**Published:** 2014-07-01

**Authors:** Daniel Rondelaud, Amal Titi, Philippe Vignoles, Abdeslam Mekroud, Gilles Dreyfuss

**Affiliations:** 1 INSERM 1094, Faculties of Medicine and Pharmacy, Limoges 87025, France; 2 PADESCA Laboratory, Veterinary Science Institute, University Constantine 1, El Khroub 25100, Algeria

**Keywords:** Cercaria, Experimental infection, *Fasciola hepatica*, *Galba truncatula*, *Lymnaea fuscus*, Prevalence, *Radix balthica*, Redia

## Abstract

**Background:**

High prevalence of *Fasciola hepatica* infection (>70%) was noted during several outbreaks before the 2000s in several French farms where *Galba truncatula* is lacking. Other lymnaeids such as *Lymnaea fuscus, L. glabra* and/or *Radix balthica* are living in meadows around these farms but only juvenile snails can sustain complete larval development of *F. hepatica* while older snails were resistant. The low prevalence of infection (<20%) and limited cercarial production (<50 cercariae per infected snail) noted with these juveniles could not explain the high values noted in these cattle herds. As paramphistomosis due to *Calicophoron daubneyi* was not still noted in these farms, the existence of another mode of infection was hypothesized. Experimental infection of several successive generations of *L. glabra*, originating from eggs laid by their parents already infected with this parasite resulted in a progressive increase in prevalence of snail infection and the number of shed cercariae. The aim of this paper was to determine if this mode of snail infection was specific to *L. glabra*, or it might occur in other lymnaeid species such as *L. fuscus* and *R. balthica.*

**Methods:**

Five successive generations of *L. fuscus* and *R. balthica* were subjected to individual bimiracidial infections in the laboratory. Resulting rediae and cercariae in the first four generations were counted after snail dissection at day 50 p.e. (20°C), while the dynamics of cercarial shedding was followed in the F5 generation.

**Results:**

In the first experiment, prevalence and intensity of *F. hepatica* infection in snails progressively increased from the F1 (*R. balthica*) or F2 (*L. fuscus*) generation. In the second experiment*,* the prevalence of *F. hepatica* infection and the number of shed cercariae were significantly lower in *L. fuscus* and *R. balthica* (without significant differences between both lymnaeids) than in *G. truncatula*.

**Conclusion:**

The *F. hepatica* infection of several successive snail generations, coming from parents infected with this parasite, resulted in a progressive increase in prevalence and intensity of snail infection. This may explain high prevalence of fasciolosis noted in several cattle-breeding farms when the common snail host of this digenean, *G. truncatula*, is lacking.

## Background

Different species of Lymnaeidae can act as intermediate hosts in the *Fasciola hepatica* life cycle. In their review, Torgerson and Claxton [1] provided a list mentioning about 20 snail species. However, all these species do not have the same capacity to sustain complete larval development of the parasite. In Western Europe, for example, *Galba truncatula* is considered as the main snail host for *F. hepatica*[1-3]. Other species living in these regions, i.e. *Lymnaea fuscus*, *L. glabra*, *L. palustris, L. stagnalis*, *Radix balthica* and *R. labiata*, can also sustain complete larval development of the parasite with cercarial shedding if they are infected by miracidia in their first weeks of life, i.e. for a shell height less than 2 mm [4]. However, experimental infections made by our team on these juvenile snails with *F. hepatica* demonstrated a strong mortality among exposed snails (often, > 80%), a low prevalence of *F. hepatica* infection (generally, < 20%) and a low number of shed cercariae [5-10]. Under these conditions, it is difficult to understand the mechanism of snail infection with *F. hepatica* when *G. truncatula* is lacking in 21 cattle-breeding farms located in central France and known for high risks of animal fasciolosis (prevalence, > 70%) before the 2000s (D. Rondelaud, personal observation). As other lymnaeid species such as *L. glabra* are living in meadows present in these farms, the existence of another mode of snail infection with *F. hepatica* to explain high prevalence of bovine fasciolosis was hypothesized.

As Chipev *et al*. [11] in the model *G. truncatula*/*F. hepatica* and Southgate *et al*. [12] in the model *Bulinus tropicus*/*Schistosoma bovis* have demonstrated the role of a paramphistomid in these snail infections, a second mode of snail infection was demonstrated for French lymnaeids other than *G. truncatula*. Using a first exposure of snails with *Calicophoron daubneyi* and a second exposure with *F. hepatica* 4 hours later, successful infections of pre-adult *L. glabra* (shell height at exposure, 2.1-4 mm) with *F. hepatica, C. daubneyi,* or both, were reported under laboratory conditions [13,14]. This result was verified in the field because some *L. glabra* and *Lymnaea palustris* infected with *F. hepatica*, *C. daubneyi*, or both, were found in swampy meadows and wild watercress beds from central or eastern France [15-18]. However, this mode of snail infection using a paramphistomid before the penetration of *F. hepatica* miracidia did not explain all the cases of bovine fasciolosis in farms where *G. truncatula* is lacking. As paramphistomosis was not still detected before the 2000s in the livestock of seven farms (D. Rondelaud, personal observation), it was necessary to suppose a third mode of snail infection with *F. hepatica* in these farms.

In natural habitats colonized by *L. glabra*[6] and *L. palustris*[7], experimental introduction of *F. hepatica* miracidia during four successive years had induced a progressive increase in prevalence of snail infection. This finding was at the origin of experiments carried out to demonstrate the existence of this third mode of snail infection for lymnaeids other than *G. truncatula*. The infection of five successive generations of pre-adult *L. glabra* (snail height at exposure, 4 ± 0.1 mm), originating from parents already infected with this parasite, resulted in a progressive increase in prevalence of snail infection and the number of shed cercariae [19]. In view of this last result, it was useful to determine if this mode of snail infection was specific to *L. glabra*, or it might occur in other lymnaeid species such as *L. fuscus* and *R. balthica* which were living with *G. truncatula* in swampy meadows of central France [20,21]. To verify either possibility, five successive generations of *L. fuscus* and *R. balthica* were subjected to individual bimiracidial infections in the laboratory. Resulting rediae and cercariae in the first four generations were counted after snail dissection at day 50 post-exposure (p.e.) at 20°C, while the dynamics of cercarial shedding was followed in the F5 generation.

## Methods

### Snails and parasite

Both snail populations were living on the commune of Thenay, department of Indre, central France. *Lymnaea fuscus* inhabited a small pond (46°35’38” N, 1°27’10” E), whereas *R. balthica* was colonizing a brook (47°23’22” N, 1°17’31” E) in the centre of Thenay. The identification of the first population as *L. fuscus* and the second as *R. balthica* was confirmed by the study of ITS-2 sequences [22,23]. Previous investigations in adults (30 per sample and per population) over the six months preceding the beginning of the experiments have demonstrated the absence of natural trematode infections in snails when they were dissected in the laboratory. One hundred adults (16–20 mm in shell height) were collected from each population and placed in 5-L and 10-L covered aquaria with 5 snails per litre of permanently-oxygenated spring water. Aquaria were subjected to constant conditions (temperature, 20° ± 1°C; light/dark period, 12 h/12 h). Dissolved calcium concentration in spring water was 35 mg/L. Pesticide-free leaves of fresh lettuce were given as food ad libitum and spring water in aquaria was changed weekly. Egg masses laid by these parents were placed into small rearing aquaria with oxygenated spring water and finely powdered lettuce. Four-millimetre high individuals were considered as the F1 generation and were used for experiments. The protocol was similar for the F2, F3, F4 and F5 snail generations (see below). A total of 300 *L. fuscus* and 300 *R. balthica*, measuring 4 ± 0.1 mm in height and belonging to the F1, F2, F3, F4 and F5 generations (Tables 1 and 2), were used for experiments.

**Table 1:** **Several characteristics of *Fasciola hepatica *** **infection in four generations of *Lymnaea fuscus *** **and *Radix balthica *** **subjected to individual bimiracidial exposures, raised at 20**°C **and dissected at day 50 post-exposure (first experiment)**

**Snail population and generation**	**Number of surviving snails at day 30 p.e. (%)**	**Number of infected snails with**				**Overall prevalence of infection (%)**
		**Immature rediae**	**Cercariae-containing rediae**	**Free cercariae**	**Cercarial shedding**	
*Lymnaea fuscus*						
- F1	16 (32.0)	-	-	-	-	-
- F2	24 (48.0)	1	-	-	-	4.1
- F3	27 (54.0)	2	2	1	-	18.5
- F4	29 (58.0)	2	4	3	1	34.4
*Radix balthica*						
- F1	26 (42.0)	2	1	-	-	11.52
- F2	27 (54.0)	2	3	1	-	22.2
- F3	33 (66.0)	1	5	2	1	27.2
- F4	31 (62.0)	2	3	7	3	48.3

p.e., post-exposure.

**Table 2:** **Characteristics of *Fasciola hepatica *** **infection in *Lymnaea fuscus *** **(F5 generation), *Radix balthica *** **(F5 generation) and *Galba truncatula *** **subjected to individual bimiracidial exposures and raised at 20**°C **(second experiment)**

**Population**	***Lymnaea fuscus***	***Radix balthica***	***Galba truncatula***	Significant differences*
Number of snails:				
- at exposure	100	100	100	
- at day 30 p.e. (%)	54 (54.0)	65 (65.0)	74 (74.0)	*χ*^2^ = 8.74, *p* < 0.05
Number of snails:				
- CS	7	13	43	
- NCS	12	14	11	
Prevalence of infection (%)	35.1	41.5	72.9	*χ*^2^ = 21.99, *p* < 0.001
Shell growth (mm):				
- CS	7.7 ± 1.5	6.0 ± 1.1	3.7 ± 0.9	*F* = 14.32, *p* < 0.001
- NCS	7.3 ± 1.1	5.7 ± 0.8	3.5 ± 1.1	*F* = 16.63, *p* < 0.001
Prepatent period in days	55.7 ± 6.5	57.1 ± 8.3	43.3 ± 5.2	*F* = 4.42, *p* < 0.05
Patent period in days	11.2 ± 4.7	16.1 ± 5.2	28.8 ± 7.3	*F* = 10.21, *p* < 0.001
Number of shed cercariae	80.3 ± 35.8	66.8 ± 21.2	157.1 ± 55.2	*F* = 7.10, *p* < 0.01
Cadavers of NCS snails:				
- Free rediae	15.7 ± 5.2	18.3 ± 4.5	31.5 ± 5.3	*F* = 11.71, *p* < 0.001
- Free cercariae	137.6 ± 37.0	154.2 ± 52.9	236.5 ± 67.1	*F* = 4.68, *p* < 0.05
Percentage of shed cercariae**	58.3	43.3	66.4	

*Mean values are given with their standard deviations for seven parameters. Each statistical comparison was performed using values recorded for the three lymnaeid species. CS, cercariae-shedding snails; *F*, value of ANOVA; NCS, non cercarial shedding snails containing cercariae; NS, non significant difference; *p*, probability; p.e., post-exposure; *χ*^2^, value of the *χ*^2^ test.

**Number of cercariae shed by CS snails / overall cercarial production in NCS snails.

To compare the characteristics of *F. hepatica* infection in *L. fuscus* and *R. balthica* with those found in a common intermediate host of this parasite, a wild population of *G. truncatula* was selected*.* This species is the main snail host of *F. hepatica* in Europe [1-3] and may be infected at any age [24]. These snails were colonizing a road ditch at Chézeau Chrétien (46°40’27” N, 1°21’21” E), commune of Chitray, department of Indre, central France. As for *L. fuscus* and *R. balthica*, no trematode larval forms were found in adult snails collected over a 6-month period before the beginning of the experiments and dissected in the laboratory. One hundred snails (shell height, 4 ± 0.1 mm), belonging to the overwintering generation, were collected and acclimatized for 24 hours to laboratory temperature before being exposed to *F. hepatica* miracidia. A single generation of *G. truncatula* was used for this study because prevalence of *F. hepatica* infection and the number of shed cercariae were significantly lower in the F1 generation born to already infected parents than those noted for the F1 generation born to unexposed parents [25].

Several isolates of *F. hepatica* eggs were regularly collected from the gall bladders of naturally infected limousine cattle at the Limoges slaughterhouse. They were washed several times with spring water and immediately incubated in the dark at 20°C for 20 days to allow miracidial development [26].

### Experimental protocol

The *L. fuscus* and *R. balthica* susceptibility to *F. hepatica* miracidia was studied during five successive snail generations via a protocol already used by Sanabria *et al.*[27] for *F. hepatica* and Vignoles *et al.*[28] for *Fascioloides magna*. F2 snails originated from eggs laid by the 50 exposed individuals of the F1 generation between weeks 2 and 5 p.e. A similar protocol was used for the F3, F4 and F5 generations. This protocol was chosen in order that these descendants have a first (F2) or multiple contacts (the F3-F5 generations) with the parasite through their infected parents.

The aim of the first experiment was to determine the aptitude of *L. fuscus* and *R. balthica* as a snail host for *F. hepatica.* Four groups of 50 *L. fuscus* each (1 group per generation from F1 to F4) were constituted. Four groups of *R. balthica* were also constituted according to the same protocol*.* Each lymnaeid was routinely exposed to two miracidia in a 35-mm Petri dish for 4 hours at 20°C (in 3.5 mL spring water). The choice of two miracidia per snail was based on the fact that a single miracidium (out of two) only developed in 75% of *G. truncatula* after exposure [29,30]. Each Petri dish was controlled 4 hours after the introduction of miracidia to verify their absence in spring water and thereby confirm their penetration into the lymnaeid. Snails were then raised in groups of 50 individuals in 10-L aquaria according to the same protocol used for parents. At day 50 p.e., surviving snails were dissected under a stereomicroscope to detect the presence of *F. hepatica* larval forms within their bodies and determine the most developed stage (immature rediae, cercariae-containing rediae, or free cercariae). The existence of cercarial shedding was also taken into account. Infected snails were then counted in relation to snail generation and each developmental stage of larval development.

As cercarial *F. hepatica* shedding (Table 1) was noted from F3 (*R. balthica*) or F4 (*L. fuscus*) generations, a second experiment was carried out to study cercarial shedding in the F5 generations and compare it with that occurring in *G. truncatula*. One hundred *L. fuscus*, 100 *R. balthica* (both belonging to the F5 generation) and 100 *G. truncatula* were used. Exposure of the three lymnaeids to miracidia and breeding of *L. fuscus* and *R. balthica* during the first 30 days were similar to those used in the first experiment. In contrast, the *G. truncatula* were raised in groups of ten individuals in 14-cm Petri dishes (60 mL spring water per dish) for 30 days according to Rondelaud *et al.*[31]. Snail food for *G. truncatula* consisted of dried lettuce leaves and dead *Molinia caerulea* leaves, while stems of live *Fontinalis* sp. ensured oxygenation of the water layer. Dissolved calcium in spring water also was 35 mg/L. Petri dishes containing *G. truncatula* were placed in the same air-conditioned room as the aquaria with *L. fuscus* or *R. balthica*. At day 30 p.e., each surviving snail was isolated in a 50-mm Petri dish containing 10 mL spring water, with pieces of dead grass, lettuce and spring moss. These Petri dishes were also placed at 20°C. Spring water and food, if necessary, were changed daily until snail death. When the first cercarial shedding occurred, surviving snails were subjected to a thermal shock every three days by placing their Petri dishes at 10°-13°C for 3 hours to stimulate cercarial exit [27]. Shed cercariae were then counted and removed from Petri dishes. At the death of each infected snail, its shell was measured using callipers. Cadavers of non cercarial shedding (NCS) snails that contained cercariae were routinely dissected under a stereomicroscope to count free rediae and free cercariae.

### Parameters studied and data analysis

The first two parameters were snail survival at day 30 p.e. and the prevalence of *F. hepatica* infection (calculated in relation to the number of snails surviving at day 30 p.e.). In the first experiment, prevalence took into account the numbers of snails with immature rediae only, cercariae-containing rediae, or with free cercariae. In the second experiment, prevalence was calculated using the numbers of cercariae-shedding (CS) and NCS snails. For each parameter, the differences were analyzed using a *χ*^2^ test. In the second experiment, shell growth of CS and NCS snails during the experiment (calculated using the difference between shell heights at exposure and at snail death), length of the prepatent and patent periods, and the number of shed cercariae were also considered. Free rediae and free cercariae counted in NCS snail cadavers were also taken into account. Individual values recorded for these last six measurements were averaged and their standard deviations were established for each snail group. One-way analysis of variance (ANOVA) was used to establish levels of statistical significance. The different analyses were performed using Statview 5.0 software (SAS Institute Inc., Cary, NC, USA).

## Results

### *Aptitude of* Lymnaea fuscus *and* Radix balthica *for* Fasciola hepatica *infection*

Table 1 gives the numbers of infected snails in F1 to F4 generations of both lymnaeids (first experiment). Survival rates of *L. fuscus* at day 30 p.e. significantly increased (*χ*^2^ = 12.66, *p* < 0.05) with increasing snail generation. The same finding was also noted for the number of snails containing live *F. hepatica* larval forms so that prevalence of infection was significantly higher (*χ*^2^ = 12.59, *p* < 0.05) in the F4 than in the other generations. In the case of *R. balthica*, the differences between survival rates at day 30 p.e. were insignificant, whatever the mode of comparison. In contrast, overall prevalence of *F. hepatica* infection significantly increased (*χ*^2^ = 10.29, *p* < 0.05) with increasing snail generation. Four successive generations of snails (*L. fuscus*) or three (*R. balthica*) were thus necessary to obtain the first cercarial shedding from an infected snail.

### *Characteristics of* Fasciola hepatica *infection*

Table 2 gives the main characteristics of *F. hepatica* infection in the three lymnaeids (second experiment). Compared to *G. truncatula*, snail survival at day 30 p.e., prevalence of *F. hepatica* infection, the patent period, and the number of shed cercariae were significantly lower in *L. fuscus* and *R. balthica*, while the prepatent period was significantly longer. No significant difference between *L. fuscus* and *R. balthica* was found, whatever the above parameter considered. Compared to *G. truncatula,* the shell growths of CS and NCS snails during the experiment were significantly greater in *L. fuscus* and *R. balthica*. In NCS snails, free rediae and free cercariae of *F. hepatica* were significantly more numerous in *G. truncatula* than in the other two lymnaeids*.* If the number of shed cercariae noted in CS snails was compared to the quantity of free cercariae recorded in the bodies of NCS snails, the percentage of shed cercariae was greater in *G. truncatula* than in the other lymnaeids (66% vs 43-58%).

## Discussion

In northern Correze, Creuse and Haute Vienne (central France), several lymnaeid species, i.e. *L. fuscus*, *L. glabra*, *L. palustris* and/or *R. balthica*, may live with *G. truncatula* along the same open drainage network in swampy meadows [20] or in the same wild watercress beds [32,33]. The presence of the former lymnaeids had raised a serious problem in several cattle-breeding farms known for high prevalence of fasciolosis when *G. truncatula* was lacking. Indeed, all the experimental infections carried out by our team with 4-mm high *L. fuscus*, *L. glabra*, *L. palustris* or *R. balthica,* and *F. hepatica* over the past 40 years were negative. On the other hand, successful infections of juvenile snails [5,8,9,34-36] were insufficient to explain high prevalence values noted in the livestock of these farms because of the strong mortality of juveniles during their infection and the low number of shed cercariae. Under these conditions, the lymnaeid species responsible for fasciolosis transmission was often difficult to identify and control. The finding of infected *L. glabra*[19], *L. fuscus* and *R. balthica* (the present study) through several successive generations of descendants coming from parents already infected with *F. hepatica* suggests a convincing explanation to farmers for incriminating snail species. However, the presence of the parasite throughout the year is necessary to have such successful infections of preadult snails and this raises again the problem of the responsible definitive host. Indeed, local cattle present in these farms were treated twice a year with anthelminthics (triclabendazole since 1990–1994) and regularly moved on new pastures for grazing. The role of local lagomorphs cannot be excluded in fasciolosis transmission because hares and rabbits were often found to be infected with *F. hepatica* in northern Haute Vienne [37].

The results noted for *L. fuscus* in the present study cannot be compared to the literature because this species had often been synonymized with *L. palustris* in the past [38], and experimental infections of *L. fuscus* with *F. hepatica* were only successful for juveniles measuring less than 2 mm at miracidial exposure [8,39]. Contrary to *L. fuscus*, several reports [3,8,40-43] described the successful infection of *Radix* species other than *R. auricularia* when juveniles were experimentally exposed to *F. hepatica*. But identification of these *Radix* species using morphological criteria was often difficult to do and was currently based on the analysis of 18S, ITS-1, ITS-2 and/or COI sequences [44-49]. As a consequence, it is difficult to compare the above results if the *Radix* populations concerned by these studies are not identified using DNA-based analyses. As the names of these *Radix* species had also changed from the 2000s [44,45], the only paper mentioning the successful infection of *R. balthica* (=*R. peregra* O.F. Müller, 1774; = *R. ovata* Draparnaud, 1805) and *R. labiata* (=*R. peregra* sensu Ehrmann, 1933) was reported in Belgium by Caron *et al*. [50,51]. According to these authors, juveniles of both species, once exposed to individual bimiracidial infections with *F. hepatica*, could sustain complete larval development of the parasite with cercarial emergence (*R. labiata*) or without shedding (*R. balthica*).

In view of this situation mainly due to the above-mentioned taxonomic problems within the Lymnaeidae family, the results noted for *L. fuscus* and *R. balthica* can only be compared to those reported by Rondelaud *et al*. [19] for two populations of *L. glabra* infected with *F. hepatica* and raised according to the same protocol. In the three lymnaeids, prevalence of infection significantly increased with increasing generation of snails, even if the speed of this process over time varied with each lymnaeid species or each population in the case of *L. glabra*. A similar finding can also be noted for the intensity of *F. hepatica* infection, expressed by the number of shed cercariae in CS snails or the quantities of free rediae and free cercariae noted in dissected NCS snails. As the values of these last parameters were significantly lower in the F5 generation of *L. fuscus* and *R. balthica* (the present study) or the F7 generation of *L. glabra*[19] than in *G. truncatula*, this suggests a progressive adaptation between each lymnaeid and the parasite though the infection of several successive snail generations, as suggested by Boray [3] in his review. However, the present study does not allow us to determine the real level of *L. fuscus* and *R. balthica* susceptibility to the parasite because our infections were only carried out through five snail generations and the answer to this question would need the infection of a greater number of generations. Two perhaps complementary hypotheses may be proposed to comment on these changes in successive generations of infected *L. fuscus* and *R. balthica*. The first would be to admit decreased genetic variation in host resistance through selection of snails with lowest resistance and/or a series of bottlenecks during the five successive generations of snails. This first hypothesis is based on the protocol used for the two lymnaeids, with the selection of offspring coming from already infected parents. However, another hypothesis, based on a progressive decrease in the number of the snail ganglionic neurons through the successive generations of infected snails originating from already infected parents, cannot be excluded. This interpretation is supported with the report by Szmidt-Adjidé *et al.*[52] on the numbers of neurons in the model *G. truncatula/F. hepatica*. According to these authors, many neurons in the dorsal lobes of cerebroid ganglia and in pedal ganglia of *G. truncatula* disappeared during snail infection and lost their function. If this neuronal death also exists in the corresponding ganglia of infected *L. fuscus* and *R. balthica*, the surviving neurons would gradually secrete a lower quantity of neuromediators [53] through successive generations of infected snails. As a consequence, this decrease could induce a progressive change in the mechanisms of the snail immune system response to *F. hepatica* and also a progressive lifting of bottleneck exerted by the snail on larval development of the parasite.

## Conclusion

The *F. hepatica* infection of several successive generations of snails, coming from parents infected with this parasite, resulted in a progressive increase in prevalence and intensity of snail infection. This mode of snail infection may explain high prevalence of fasciolosis noted in several cattle-breeding farms when the common snail host of this digenean, *G. truncatula*, was lacking.

## Competing interests

The authors declare that they have no competing interests.

## Authors’ contribution

DR, AT and AM designed the study, participated in field collection, performed all laboratory work and wrote main parts of the manuscript. PV made statistical analyses. GD conceived the study and contributed to the interpretation of data and writing of the manuscript. All authors have read and approved the final manuscript.

## References

[B1] Torgerson P Claxton J Dalton JP Epidemiology and control Fasciolosis 1999 Oxon: CABI Publishing 113 149

[B2] Taylor EL Fascioliasis and the liver-fluke 1965 64 Rome: FAO Agricultural Studies

[B3] Boray JC Experimental fascioliasis in Australia Adv Parasitol 1969 7 95 210 4935272 10.1016/s0065-308x(08)60435-2

[B4] Boray JC The potential impact of exotic *Lymnaea* spp. on fascioliasis in Australasia Vet Parasitol 1978 4 127 141 10.1016/0304-4017(78)90004-3

[B5] Busson P Busson D Rondelaud D Pestre-Alexandre M Données expérimentales sur l'infestation des jeunes de cinq espèces de limnées par *Fasciola hepatica* L Ann Parasitol Hum Comp 1982 57 555 563 7168528

[B6] Bouix-Busson D Rondelaud D L'infestation de *Lymnaea glabra* Müller par *Fasciola hepatica* L. Etude expérimentale sur le terrain Ann Parasitol Hum Comp 1986 61 215 225 6638788

[B7] Dreyfuss G Moukrim A Rondelaud D Vareille-Morel C Several field observations concerning infection of *Lymnaea palustris* by *Fasciola hepatica* J Helminthol 1994 68 115 118 10.1017/S0022149X00013626 7930451

[B8] Dreyfuss G Abrous M Rondelaud D The susceptibility of *Lymnaea fuscus* to experimental infection with *Fasciola hepatica* J Parasitol 2000 86 158 160 10701582 10.1645/0022-3395(2000)086[0158:TSOLFT]2.0.CO;2

[B9] Dreyfuss G Vignoles P Rondelaud D Variability of *Fasciola hepatica* infection in *Lymnaea ovata* in relation to snail population and snail age Parasitol Res 2000 86 69 73 10.1007/s004360050013 10669140

[B10] Vignoles P Dreyfuss G Rondelaud D Redial growth and cercarial productivity of *Fasciola hepatica* in three species of young lymnaeid snails J Helminthol 2002 76 269 272 10.1079/JOH2002118 12363381

[B11] Chipev N Vassilev I Samnaliev P **Interactions between *****Paramphistomum cf daubneyi Dinnik***, **1962 and *****Fasciola hepatica L*****. in successive cross-invasions of *****Lymnaea (Galba) truncatula.*** Helminthologia 1985 20 80 88

[B12] Southgate VR Brown DS Warlow R Knowles RJ Jones A The influence of *Calicophoron microbothrium* on the susceptibility of *Bulinus tropicus* to *Schistosoma bovis* Parasitol Res 1989 75 381 391 10.1007/BF00931134 2726720

[B13] Abrous M Rondelaud D Dreyfuss G *Paramphistomum daubneyi* and *Fasciola hepatica*: the effect of dual infection on prevalence and cercarial shedding in preadult *Lymnaea glabra* J Parasitol 1996 82 1026 1029 10.2307/3284217 8973417

[B14] Abrous M Rondelaud D Dreyfuss G Cabaret J Unusual transmission of the liver fluke, *Fasciola hepatica*, by *Lymnaea glabra* or *Planorbis leucostoma* in France J Parasitol 1998 84 1257 1259 10.2307/3284683 9920323

[B15] Abrous M Rondelaud D Dreyfuss G Cabaret J Infection of *Lymnaea truncatula* and *Lymnaea glabra* by *Fasciola hepatica* and *Paramphistomum daubneyi* in farms of central France Vet Res 1999 30 113 118 10081118

[B16] Abrous M Rondelaud D Dreyfuss G A field study of natural infections in three freshwater snails with *Fasciola hepatica* and/or *Paramphistomum daubneyi* in central France J Helminthol 2000 74 189 194 10.1017/S0022149X00000275 10953217

[B17] Degueurce F Abrous M Dreyfuss G Rondelaud D Gevrey J *Paramphistomum daubneyi* and *Fasciola hepatica*: the prevalence of natural or experimental infection in four species of freshwater snails in eastern France J Helminthol 1999 73 197 202

[B18] Rondelaud D Cressonnières naturelles du Limousin et risques de distomatose humaine à *Fasciola hepatica* Ann Sci Limousin 2004 15 1 14 Published online in *Annales Scientifiques du Naturaliste,* 2012

[B19] Rondelaud D Djuikwo-Teukeng FF Vignoles P Dreyfuss G *Lymnaea glabra*: progressive increase in susceptibility to *Fasciola hepatica* through successive generations of experimentally-infected snails J Helmintholin press 10.1017/S0022149X14000169 24735873

[B20] Vareille-Morel C Dreyfuss G Rondelaud D The characteristics of habitats colonized by three species of *Lymnaea* in swampy meadows on acid soil: their interest for fasciolosis control Ann Limnol-Int J Limnol 1999 35 173 178 10.1051/limn/1999024

[B21] Rondelaud D Vignoles P Dreyfuss G La Limnée tronquée, un mollusque d’intérêt médical et vétérinaire 2009 Limoges: Presses Universitaires du Limousin (PULIM)

[B22] Novobilský A Kašný M Beran L Rondelaud D Höglund J *Lymnaea palustris* and *Lymnaea fuscus* are potential but uncommon intermediate hosts of *Fasciola hepatica* in Sweden Parasit Vectors 2013 6 251 10.1186/1756-3305-6-251 23985077 PMC3765860

[B23] Schniebs K Glöer P Vinarski MV Hundsdoerfer AK Intraspecific morphological and generic diversity in *Radix balthica* (Linnaeus 1758) (Gastropoda: Basommatophora: Lymnaeidae) with morphological comparison to other European *Radix* species J Conchol 2011 40 657 678

[B24] Gold D Growth and survival of the snail *Lymnaea truncatula*: effects of soil type, culture medium and *Fasciola hepatica* infection Isr J Zool 1980 29 163 170

[B25] Vignoles P Rondelaud D Dreyfuss G A first infection of *Galba truncatula* with *Fasciola hepatica* modifies the prevalence of a subsequent infection and cercarial production in the F1 generation Parasitol Res 2003 91 349 352 10.1007/s00436-003-0891-9 14574569

[B26] Ollerenshaw CB Some observations on the epidemiology of fascioliasis in relation to the timing of molluscicide applications in the control of the disease Vet Rec 1971 88 152 164 10.1136/vr.88.6.152 5102169

[B27] Sanabria R Mouzet R Courtioux B Vignoles P Rondelaud D Dreyfuss G Cabaret J Romero J Intermediate snail hosts of French *Fasciola hepatica*: *Lymnaea neotropica* and *Lymnaea viatrix* are better hosts than local *Galba truncatula* Parasitol Res 2012 111 2011 2016 10.1007/s00436-012-3049-9 22864862

[B28] Vignoles P Novobilský A Höglund J Kašný M Pankrác J Dreyfuss G Pointier JP Rondelaud R *Lymnaea cubensis* (Pfeiffer, 1839), an experimental intermediate host for *Fascioloides magna* Folia Parasitol 2014 61 185 188 24822325

[B29] Préveraud-Sindou M Dreyfuss G Rondelaud D Comparison of the migrations of *Fasciola hepatica* sporocysts in *Lymnaea truncatula* and other related snail families Parasitol Res 1994 80 342 346 10.1007/BF02351877 8073023

[B30] Préveraud-Sindou M Rondelaud D Localization and outcome of *Fasciola hepatica* sporocysts in *Lymnaea truncatula* subjected to mono- or plurimiracidial exposures Parasitol Res 1995 81 265 267 7770435 10.1007/BF00937121

[B31] Rondelaud D Fousi M Vignoles P Moncef M Dreyfuss G Optimization of metacercarial production for three digenean species by the use of Petri dishes for raising lettuce-fed *Galba truncatula* Parasitol Res 2007 100 861 865 10.1007/s00436-006-0353-2 17061111

[B32] Rondelaud D Données épidémiologiques sur la distomatose humaine à *Fasciola hepatica* L. dans la région du Limousin, France. Les plantes consommées et les limnées vectrices Ann Parasitol Hum Comp 1980 55 393 405 7458166

[B33] Rondelaud D Dreyfuss G Bouteille B Dardé ML Changes in human fasciolosis in a temperate area. About some observations over a 28-year period in central France Parasitol Res 2000 86 753 757 10.1007/PL00008563 11002984

[B34] Bouix-Busson D Rondelaud D Prevost J Influence du nombre de miracidiums et de l'âge du Mollusque sur la survie et le degré d'infestation de *Lymnaea glabra* Müller par *Fasciola hepatica* L Ann Parasitol Hum Comp 1983 58 347 352 6638788

[B35] Bouix-Busson D Rondelaud D Barthe D Experimental infection of *Lymnaea glabra* and *L. truncatula* by *Fasciola hepatica* J Parasitol 1984 70 1002 1003 10.2307/3281665 6512654

[B36] Bouix-Busson D Rondelaud D Combes C L'infestation de *Lymnaea glabra* Müller par *Fasciola hepatica* L. Les caractéristiques des émissions cercariennes Ann Parasitol Hum Comp 1985 60 11 21

[B37] Rondelaud D Vignoles P Abrous M Dreyfuss G The definitive and intermediate hosts of *Fasciola hepatica* in the natural watercress beds in central France Parasitol Res 2001 87 475 478 10.1007/s004360100385 11411948

[B38] Jackiewicz M Błotniarki Europy (Gastropoda: Pulmonata: Lymnaeidae) 2000 Poznan: Wydawnictwo Kontekst

[B39] Novobilský A Kašný M Pankrác J Rondelaud D Engström A Höglund J *Lymnaea fuscus* (Pfeiffer, 1821) as a potential intermediate host of *Fascioloides magna* in Europe Exp Parasitol 2012 132 282 286 10.1016/j.exppara.2012.08.005 22902745

[B40] Kendall SB Snail hosts of *Fasciola hepatica* in Britain J Helminthol 1950 24 63 74 10.1017/S0022149X00019131

[B41] Berghen P Some Lymnaeidae as intermediate hosts of *Fasciola hepatica* in Belgium Exp Parasitol 1964 15 118 124 10.1016/0014-4894(64)90012-8 14167545

[B42] Boray JC Studies on the relative susceptibility of some lymnaeids to infection with *Fasciola hepatica* and *F. gigantica* and on the adaptation of *Fasciola* spp Ann Trop Med Parasitol 1966 60 114 124 5960101 10.1080/00034983.1966.11686394

[B43] Sindou P Rondelaud D Barthe D *Fasciola hepatica* L.: étude comparative de la pathologie viscérale chez six espèces de limnées soumises dès leur naissance à des infestations monomiracidiennes individuelles Bull Soc Zool Fr 1990 115 331 340

[B44] Bargues MD Vigo M Horak P Dvorak J Patzner RA Pointier JP Jackiewicz M Meier-Brook C Mas-Coma S European Lymnaeidae (Mollusca: Gastropoda), intermediate hosts of trematodiases, based on nuclear ribosomal DNA ITS-2 sequences Infect Genet Evol 2001 1 85 107 10.1016/S1567-1348(01)00019-3 12798024

[B45] Bargues MD Horak P Patzner RA Pointier JP Jackiewicz M Meier-Brook C Mas-Coma S Insights into the relationships of Palearctic and Nearctic lymnaeids (Mollusca: Gastropoda) by rDNA ITS-2 sequencing and phylogeny of stagnicoline intermediate host species of *Fasciola hepatica* Parasite 2003 10 243 255 14535164 10.1051/parasite/2003103243

[B46] Bargues MD Mas-Coma S Reviewing lymnaeid vectors of fascioliasis by ribosomal DNA sequence analyses J Helminthol 2005 79 257 267 10.1079/JOH2005297 16153320

[B47] Mas-Coma S Valero MA Bargues MD *Fasciola*, lymnaeids and human fascioliasis, with a global overview on disease transmission, epidemiology, evolutionary genetics, molecular epidemiology and control Adv Parasitol 2009 69 41 146 19622408 10.1016/S0065-308X(09)69002-3

[B48] Correa AC Escobar JS Durand P Renaud F David P Jarne P Pointier JP Hurtrez-Bousses S Bridging gaps in the molecular phylogeny of the Lymnaeidae (Gastropoda: Pulmonata), vectors of fascioliasis BMC Evol Biol 2010 10 381 10.1186/1471-2148-10-381 21143890 PMC3013105

[B49] Correa AC Escobar JS Noya O Velasquez LE Gonzalez-Ramirez C Hurtrez-Bousses S Pointier JP Morphological and molecular characterization of Neotropic Lymnaeidae (Gastropoda: Lymnaeoidea), vectors of fasciolosis Infect Genet Evol 2011 11 1978 1988 10.1016/j.meegid.2011.09.003 21968212

[B50] Caron Y Lasri S Losson B *Fasciola hepatica*: an assessment on the vectorial capacity of *Radix labiata* and *R. balthica* commonly found in Belgium Vet Parasitol 2007 149 95 103 10.1016/j.vetpar.2007.07.012 17697752

[B51] Caron Y Martens K Lempereur L Saegerman C Losson B New insight in lymnaeid snails (Mollusca, Gastropoda) as intermediate hosts of *Fasciola hepatica* (Trematoda, Digenea) in Belgium and Luxembourg Parasit Vectors 2014 7 66 10.1186/1756-3305-7-66 24524623 PMC3926267

[B52] Szmidt-Adjidé V Rondelaud D Dreyfuss G Cabaret J The effect of parasitism by *Fasciola hepatica* and *Muellerius capillaris* on the nerve ganglia of *Lymnaea truncatula* J Invertebr Pathol 1996 67 300 305 10.1006/jipa.1996.0045 8812611

[B53] de Jong-Brink M How trematode parasites interfere with reproduction of their intermediate hosts, freshwater snails J Med Appl Malacol 1990 2 101 133

